# Antibiotic Use and Fatal Outcomes among Critically Ill Patients with COVID-19 in Tacna, Peru

**DOI:** 10.3390/antibiotics10080959

**Published:** 2021-08-09

**Authors:** Cesar Copaja-Corzo, Miguel Hueda-Zavaleta, Vicente A. Benites-Zapata, Alfonso J. Rodriguez-Morales

**Affiliations:** 1 Faculty of Health Sciences, Universidad Privada de Tacna, Tacna 23003, Peru; cescopajac@upt.pe; 2 Hospital III Daniel Alcides Carrión EsSalud, Tacna 23000, Peru; 3 Unidad de Investigación para la Generación y Síntesis de Evidencias en Salud, Universidad San Ignacio de Loyola, Lima 15024, Peru; vbenites@usil.edu.pe; 4 Master Program on Clinical Epidemiology and Biostatistics, Universidad Cientifica del Sur, Lima 15046, Peru; 5 Grupo de Investigación Biomedicina, Faculty of Medicine, Fundación Universitaria Autónoma de las Américas, Belmonte, Pereira, Risaralda 660003, Colombia

**Keywords:** SARS-CoV-2, COVID-19, mortality, hospital mortality, cross-infection, infections, anti-bacterial agents, drug resistance, bacterial, Peru

## Abstract

Overuse of antibiotics during the Coronavirus Disease 2019 (COVID-19) pandemic could increase the selection of extensively resistant bacteria (XDR). However, it is unknown what impact they could have on the evolution of patients, particularly critically ill patients. This study aimed to evaluate the characteristics and impact of ICU-acquired infections in patients with COVID-19. A retrospective cohort study was conducted, evaluating all patients with critical COVID-19 admitted to the intensive care unit (ICU) of a hospital in Southern Peru from 28 March 2020 to 1 March 2021. Of the 124 patients evaluated, 50 (40.32%) developed a healthcare-associated infection (HAI), which occurred at a median of 8 days (IQR 6–17) after ICU admission. The proportion of patients with HAI that required ceftriaxone was significantly higher; the same was true for the use of dexamethasone. Forty bacteria isolations (80%) were classified as XDR to antibiotics, with the most common organisms being *Acinetobacter baumannii* (54%) and *Pseudomonas aeruginosa* (22%); 33% (41/124) died at the ICU during the follow-up. In the adjusted analysis, healthcare-associated infection was associated with an increased risk of mortality (aHR= 2.7; 95% CI: 1.33–5.60) and of developing acute renal failure (aRR = 3.1; 95% CI: 1.42–6.72). The incidence of healthcare infection mainly by XDR pathogens is high in critically ill patients with COVID-19 and is associated with an increased risk of complications or death.

## 1. Introduction

The Coronavirus Disease 2019 (COVID-19) outbreak was declared a pandemic in early 2020. Since then, its etiological agent, the Severe Acute Respiratory Syndrome Coronavirus 2 (SARS-CoV-2), has spread worldwide. This pandemic has created an unprecedented challenge for healthcare systems [1], mainly because of the large number of patients requiring intensive care unit (ICU) management, despite the low proportion of severe patients [2]. It should be remembered that as of 31 July 2021, more than 197.5 million cases of COVID-19 have been diagnosed (and 4.21 million deaths recorded).

A large number of patients with COVID-19 admitted to the ICU require invasive measures, including mechanical ventilation, central venous catheters, and foley catheters, among others, which may increase the risk of developing healthcare-associated infections [3]. These secondary infections have represented a significant cause of morbidity and mortality in other pandemics such as influenza [4]. However, the impact of these infections on mortality in patients with COVID-19 and its association with the use of antibiotics, especially in Peru and Latin America, are still uncertain.

In addition, recent studies have reported an unwarranted increase in the use of antibiotics in patients with COVID-19, especially in Latin America and particularly in Peru, where it is reported that up to 99% of patients received antibiotics at hospitalization [5,6]. Moreover, antibiotics are easily sold without a medical prescription, facilitating their inappropriate use and consequences. Therefore, the impact of overuse of these therapies on the spread of bacterial resistance is a matter of concern, which may be worse during the COVID-19 pandemic, where multiple antimicrobials (e.g., azithromycin) have been repurposed, without evidence supporting its clinical benefit as antiviral in the SARS-CoV-2 infection. Indeed, the correct use of antibiotics in COVID-19 is critical for secondary infections due to bacteria, especially in the respiratory tract. Therefore, the primary objective of this study was to assess the impact of healthcare-associated infection on mortality in patients with COVID-19 admitted to the ICU of a referral hospital in Southern Peru, one of the countries with the highest COVID-19 mortality rate in the world [7] in 2021.

## 2. Results

### 2.1. Patients

**Figure 1:**
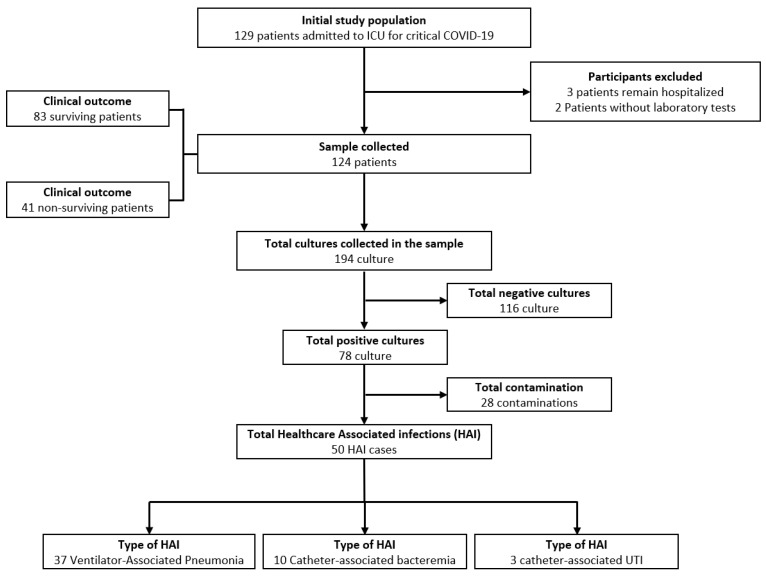
Sample selection flow chart.

During the study period, 129 patients with critical COVID-19 required invasive mechanical ventilation and were admitted to the ICU. Five patients were excluded (three were still in the ICU at the end of the study, and two did not have all the laboratory tests). The flow diagram of the study population is shown in Figure 1. A total of 124 adults hospitalized for critical COVID-19 were analyzed. From the total, 82.26% (*n* = 102) were male, with a mean age of 54.47 years (standard deviation, SD ± 12.03); their median hospital and ICU stay durations were 19 days (interquartile range, IQR 14–27.5) and ten days (IQR 6–16.5), respectively. The most frequent comorbidities were obesity (61.29%), diabetes (28.23%), and hypertension (28.23%).

During the initial emergency care, the patients’ median oxygen saturation and PaO_2_/FiO_2_ were 87% (IQR 80–89) and 249.5 (IQR 175–294), respectively. Antibiotics were administered to 95.16% (*n* = 118) of patients, the most frequent being ceftriaxone (74.19%) and azithromycin (33.06%). On admission to the ICU, median leukocytes, C-reactive protein, and procalcitonin were 10,100 cells/mm^3^ (IQR 7000–13,000), 12.52 mg/dL (IQR 6.62–17.43), and 0.12 ng/mL (IQR 0.03–0.385), respectively (Table 1).

Mortality during hospitalization was 33.06% (*n* = 41), with a mortality rate of 1.45 deaths per 100 people/days at risk. On admission to the ICU, the most frequent complications were acute respiratory distress syndrome (ARDS) (100%), sepsis (66.94%), and healthcare-associated infection (40.32%). Likewise, antibiotics were administered to 99.19% (*n* = 123) of the patients, with piperacillin/tazobactam (44.35%) and vancomycin (34.68%) being the most frequently used. In addition, 98.37% (*n* = 121) of patients received corticosteroid treatment, with methylprednisolone 100 mg being the most commonly used treatment in 54.03% (*n* = 67), followed by dexamethasone 6 mg with 37.10% (*n* = 46). Of the group, 48.39% (*n* = 60) received vasopressor therapy and 12.10% (*n* = 15) required renal replacement therapy (Table 2).

### 2.2. Bivariate Analysis According to Mortality in the Study Population

During the hospital stay, statistically significant differences were observed concerning mortality in patients over 60 years of age and in those with comorbidities such as hypertension, heart failure, asthma, chronic kidney disease (CKD), and immunosuppression. PaO_2_/FiO_2_ was lower in the group of patients who died (*p* = 0.002). It was also observed that the increases in procalcitonin, LDH, total CPK, and creatinine were higher in patients who died. The most frequent complications in the group of patients who died were bacterial superinfection, especially when the pathogen was XDR, sepsis, septic shock, acute kidney failure (AKI), gastrointestinal bleeding, arrhythmias, and in-hospital cardiac arrest (Table 2).

### 2.3. Source of Infection and Microbiological Characteristics

Of 50 (40.32%) episodes of healthcare-associated infection, ventilator-associated pneumonia was the most frequent, occurring in 37 patients, followed by catheter-associated bacteremia in 10 patients. The median time from ICU admission to the onset of healthcare-associated infection was eight days (IQR 6–17). (Table 2) The most frequent pathogen in ventilator-associated pneumonia (VAP) was *Acinetobacter baumannii*, in 25 (67.57%) patients, followed by *Pseudomonas aeruginosa* in seven patients. The most frequent cause of catheter-associated bacteremia was *Staphylococcus epidermidis*, in three patients, followed by *Acinetobacter baumannii* in two patients (Supplementary Table S1).

Of all the isolated bacteria, 40 (80%) were classified as XDR, the most frequent being *Acinetobacter baumannii* in 25 (62.5%) cultures and *Pseudomonas aeruginosa* in 10 (25%) cultures. In the same way, six (12%) bacterial isolates were classified as MDR, the most frequent being *Staphylococcus epidermidis* in two (33%) cultures (Supplementary Tables S2 and S3). During ICU stay, the most frequent characteristics among patients who developed healthcare-associated infection were: age over 60 years, chronic kidney disease, and prolonged hospital stay. Moreover, leukocytes, lymphocytes, procalcitonin, LDH, and total CPK were higher in this group. Conversely, a lower proportion of patients that did not develop infection required ceftriaxone as an empirical antibiotic and 6 mg dexamethasone as a corticosteroid (Table 3).

### 2.4. Survival Estimated by Kaplan–Meier Curves

**Figure 2:**
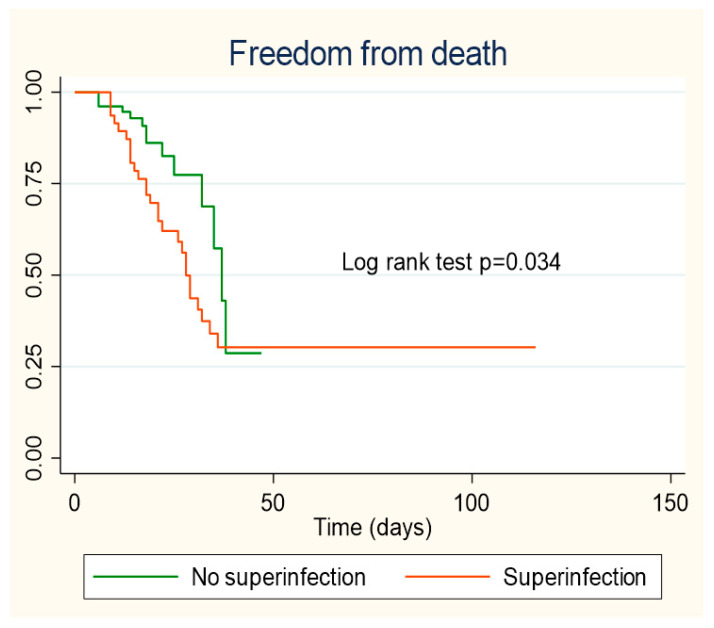
Kaplan–Meier survival curves according to healthcare-associated infection.

Patients who did not present healthcare-associated infection during hospitalization showed a better survival curve, the difference being statistically significant (*p* = 0.034) (Figure 2).

### 2.5. Healthcare Infection Associated with Mortality and Acute Renal Failure

In the crude Cox regression analysis, healthcare-associated infection was associated with an increased risk of mortality (cHR = 2.216; 95% CI 1.12–4.39). In the multivariate analysis, after adjusting for age, sex, comorbidities, and PaO_2_/FiO_2_ levels, healthcare-associated infection was independently associated with hospital mortality (aHR = 2.733; 95% CI 1.33–5.60). Likewise, PaO_2_/FiO_2_ values between 200 and 300 (aHR = 4.256; 95% CI 1.14–15.80), 100 and 200 (aHR = 3.796; 95% CI 1.01–14.47), or less than 100 (aHR = 5.503; 95% CI 1.34–22.52) were independently associated with in-hospital mortality (Table 4).

In the second analysis using crude robust variance Poisson regression, healthcare-associated infection was associated with an increased risk of acute renal failure (cRR = 3.594; 95% CI 1.60–8.05), while in the multivariate model, after adjusting for age, sex, comorbidities, and PaO_2_/FiO_2_ grades, healthcare-associated infection was independently associated with an increased risk of acute renal failure (aRR = 3.093; 95% CI 1.42–6.72) (Table 5).

## 3. Discussion

In the present retrospective cohort study in critically ill COVID-19 patients, we found a high incidence of healthcare-associated infections, primarily due to extremely antibiotic-resistant pathogens; these infections were associated with an increased risk of developing acute renal failure and significantly impacted in-hospital mortality, representing the leading cause of death in 70% of patients who died in the ICU.

The incidence of healthcare-associated infections observed in this study was high compared to that reported in other studies, where the proportion of patients with secondary infection ranged between 5% and 30% [8,9,10,11], probably due to the variability of the diagnostic methods used as well as the treatments indicated. However, when these results are compared with those of studies that only evaluated patients hospitalized in the ICU, more similar results are observed, with incidences of in-hospital infections ranging between 40% and 58% [3,12,13,14]. This high incidence, especially in patients in the ICU, could be explained by different reasons: (a) SARS-CoV-2 virulence factors could compromise the innate immune response at several levels, resulting in increased bacterial adhesion, growth, and dissemination [15]; (b) the possible anti-inflammatory or immunosuppressive effect developed by the use of steroids and biological agents (anti-IL-6 receptor monoclonal antibodies) [16]; (c) invasive procedures cause a breakdown of the body’s first defense barrier; (d) inflammatory syndromes, hypercatabolism, and physical immobilization, typical of critical patients, predispose them to nutritional alterations, which would generate a greater risk of developing secondary infections [17].

The mortality found in this study (33%) is similar to that reported for ICU patients in other hospitals [12,18,19,20]. Although most of these reports are from high-income countries, patients with critical COVID-19 in our hospital were governed by constantly updated protocols as new evidence appeared, which probably impacted mortality.

The study by Bardi, T. et al. [12] used a similar methodological structure to our study. Although there are apparent differences in the populations, treatments, and local bacterial resistance, they also found that healthcare-associated infection was independently associated with an increased risk of hospital mortality (OR 2.7; 95% CI: 1.2–5.9). Likewise, in the systematic review and meta-analysis by Musuuza, J. et al. [21], in which they included patients with superinfection or bacterial coinfection associated with CODVID-19, although 35% were case series and included studies performed outside the ICU, they found a higher probability of death in superinfected patients compared to those with only SARS-CoV-2 infection (PR 3.31; 95% CI: 1.82–5.99).

The most frequently isolated bacterial microorganisms in our cohort were similar to those reported in other studies, where the three main bacteria were *Acinetobacter spp*. (22.0%), *Pseudomonas* (10.8%), and *Escherichia coli* (6.9%) [21], although with a higher prevalence in our cohort of non-fermenting bacteria, mainly *Acinetobacter* and *Pseudomonas* in respiratory infections, which differs from that reported by other authors, where *Pseudomonas aeruginosa* (21. 1%), *Klebsiella spp.* (17.2%), and *Staphylococcus aureus* (13.5%) were the most commonly isolated microorganisms in respiratory samples [10]. This difference is particularly relevant due to the high rate of carbapenem resistance of *A. baumannii* and *Pseudomonas aeruginosa* in this cohort, which was 96% and 100%. This high proportion of multidrug-resistant microorganisms could be due to the almost universal consumption of antibiotics in our cohort (99%), well above that described by other authors (64–72%) [22,23,24], and to the deviation from adequate infection prevention and control practices, probably related to the collapse of the Peruvian health system [20].

The role of procalcitonin (PCT) in guiding antibiotic therapy in critically ill COVID-19 patients is still uncertain. Pink et al. [25] reported that those patients with COVID-19 and secondary bacterial infection during hospitalization had higher PCT values (0.4 vs. 0.1 ng/mL; *p* = 0.016) and a cut-off point of >0.55 ng/mL, which managed to discriminate those patients with secondary bacterial infection with a sensitivity and specificity of 91% and 81%. In our study, higher PCT values were also observed in those with healthcare-associated infection, although with a slightly lower cut-off point. However, in some other studies, the procalcitonin value did not show statistically significant differences in patients with bacterial superinfections [26,27]. This phenomenon is probably because, usually, the level of procalcitonin increases in the presence of cytokines, caused by a bacterial infection (IL-1b, TNF-a, and IL-6), and its production is regulated by the signal transducer and activator of transcription 3 (STAT-3). In contrast, increased IFN-gamma, often associated with viral infections, inhibits PCT production. Surprisingly, the SARS-CoV-2 virus inhibits STAT-1 and IFN function, leading to the compensatory activation of STAT3, which would be responsible for some phenomena of severe COVID-19, such as coagulopathy, thrombosis, proinflammatory state, T-cell lymphopenia, increased ACE2 expression (STAT-3 alpha), and also increased procalcitonin, which could falsely be interpreted as bacterial superinfection [28].

In order to avoid the inappropriate use of antibiotics, Foschi C. et al. [29] performed a study in which the performance of multiplex PCR (FilmArray^®^ pneumonia Plus panel) compared to traditional culture for the detection of respiratory pathogens in patients with COVID-19 hospitalized in ICU, which showed a sensitivity and specificity of 89.6% and 98.3%, respectively, and a significant decrease in the turn-around time of at least 48 h, with a high negative predictive value that could be useful for the appropriate initiation of antibiotics, ruling out bacterial superinfections and reducing unnecessary antibiotic consumption, which is why its implementation should be considered in antimicrobial stewardship programs, at least in patients with critical COVID-19.

We observed that healthcare-associated infection (HAI) was associated with an increased risk of developing acute renal failure (AKI), probably because a significant proportion of over-infected patients developed sepsis and septic shock, which would increase the hyperinflammatory state observed in patients with critical COVID-19 [30], causing circulatory collapse and reducing renal perfusion. Several studies have supported this hypothesis by examining renal biopsies, demonstrating that acute tubular injury is the most common pathological finding in patients with COVID-19 and AKI [31,32,33,34]. In addition, an acute tubular injury may occur in the context of prolonged volume depletion and in hemodynamic states that reduce renal perfusion, as is the case in over-infected patients and hyperinflammatory states [30]. This study compared those that developed HAI with those that did not, as well as also assessing the prevalence of chronic kidney disease and the hospital stay length, time at ICU, and mechanical ventilation, among other variables. As expected, the median number of leukocytes was also higher among them. Regarding the laboratory findings, procalcitonin, LDH, and total CPK were significantly higher too. Those infected required significantly more ceftriaxone, dexamethasone (6 mg), dialysis, and vasopressor therapies.

This study has some limitations. First, the study design was retrospective. Second, the number of patients evaluated was small, which reduces the possibility of controlling for various confounding and data collection and inclusion of more variables (for example, drugs administered) within the regression model. On the other hand, for the diagnosis of healthcare-associated infection, only those patients with positive conventional cultures were evaluated; therefore, it is possible that those patients with healthcare-associated infections with negative cultures due to the use of antibiotics at the time that the samples were obtained, or low bacterial load, were not included. Likewise, the laboratory data were obtained at hospital admission, and these could vary over time, especially in those with superinfection.

Furthermore, since practically all the patients received antibiotics at hospital admission, we could not evaluate their influence on superinfection and the proposed outcomes. Finally, this study was conducted in only one health institution, which has its local epidemiology of antimicrobial resistance, limiting the generalizability of these results. Therefore, prospective, multicenter studies are needed, including a more significant number of patients and a more significant amount of information about them.

## 4. Materials and Methods

### 4.1. Study Design and Setting

A retrospective cohort study was designed using data collected from a level III hospital with 110 inpatient beds and 24 intensive care beds, the Daniel Alcides Carrión Hospital. Tacna, Peru. The study period was from 28 March 2020 to 1 March 2021. The study was devised following the STROBE recommendations for reporting observational studies [35].

### 4.2. Population and Sample

The study population included all adult patients (≥18 years) who were hospitalized in the ICU for infection secondary to SARS-CoV-2, confirmed by positive nasopharyngeal swab real-time polymerase chain reaction (RT-PCR), and who also presented critical COVID-19, defined according to World Health Organization criteria by the presence of acute respiratory distress syndrome (ARDS) or sepsis or septic shock [36]. Patients were excluded when they were still in hospitalization during data collection, when they did not have all the laboratory tests, and when the diagnosis of COVID-19 could not be confirmed.

The study by Bardi, T. et al. [12] was used to find the statistical impact and the independent variable (exposure) of bacterial superinfection. In their study, 54.3% of patients with healthcare-associated infection died during follow-up, compared to 24% mortality in those without healthcare-associated infection. Likewise, an unexposed/exposed ratio of 1.45 (83/57) was reported. With these parameters, at a confidence level of 95% and 124 participants in the sample of this study, the statistical power of 93.3% was calculated. Due to the limited number of ICU beds available in the hospital (*n* = 24), we projected *a priori* that there would be a limited number of patients, so we decided to evaluate all those in the study period who met the eligibility criteria.

### 4.3. Data Collection and Variable Definition

Information was collected from the electronic medical records of the patients at the time of admission to emergency (oxygen saturation and PaFiO_2_). From when they were hospitalized in the ICU (clinical characteristics, complications and treatment) until the clinical outcome (discharge or death), the data were collected by two investigators, a double entry of data was performed (by different investigators), and a third investigator was in charge of quality control. When some variation was found, the information was contrasted with the digital medical record. In addition, cases of healthcare-associated infection (ventilator-associated pneumonia or intravascular catheter-related bacteremia or catheter-associated urinary tract infection) were reviewed by a specialist in infectious and tropical diseases to determine the presence of a true healthcare-associated infection and its origin.

#### 4.3.1. Result Variables

##### Hospital Mortality

Mortality in patients with a diagnosis of COVID-19 was assessed as an outcome variable. This was collected according to the outcome recorded in the medical record until the end of the follow-up (1 March 2021).

##### Acute Kidney Failure

Acute kidney injury (AKI) was assessed as a complication during ICU stay, diagnosed according to the Kidney Disease Improving Global Outcome (KDIGO) criteria clinical practice guidelines [37].

#### 4.3.2. Exposure Variables

##### Ventilator-Associated Pneumonia

Ventilator-associated pneumonia (VAP) was defined as a new or changing chest X-ray infiltrate or infiltrates that occurred more than 48 h after the initiation of invasive mechanical ventilation, in addition to three of the following criteria: new onset of fever (body temperature ≥ 38 °C), hypothermia (body temperature ≤ 35 °C), leukocytosis (total peripheral white blood cell count ≥ 10,000 cell/μL), leukopenia (total leukocyte count ≤ 4500 cell/μL), or left shift (>15% immature neutrophils); new respiratory aspirated secretions or need for acute changes in the ventilator support system to improve oxygenation; and positive culture for bacterial respiratory pathogens [38].

##### Intravascular Catheter-Related Bacteremia

Intravascular catheter-related bacteremia was diagnosed according to the recommendations of the clinical practice guidelines for the diagnosis and management of intravascular catheter-related infection of the Infectious Diseases Society of America (IDSA) [39].

##### Catheter-Associated Urinary Tract Infection

Catheter-associated urinary tract infection (UTI) was diagnosed if there was a positive culture with more than 103 colony-forming units/mL of uropathogenic bacteria in the presence of signs or symptoms compatible with UTI in a patient with a urinary catheter [40].

##### Bacterial Isolation, Susceptibility, and Resistance

Clinical samples were obtained during routine evaluations in the ICU and were sent to the hospital microbiology laboratory. The DL-BT64 automated detection system processed blood cultures, and when these were positive, as well as respiratory samples (endotracheal aspirate, bronchoalveolar lavage, or bronchial brushing), they were incubated for 24–48 h on blood agar or MacConkey agar; bacterial colonies were recovered and reconstituted in a 0.5 McFarland suspension (in 0.45% NaCl) and then inoculated in MicroScan Panel negative combo type 66 and Panel positive combo type 42 on the corresponding panels. Susceptibility testing and identification of bacterial isolates were performed using the Microscan Walkaway 96S^®^ plus system according to the manufacturer’s instructions.

Microorganisms were defined as multidrug-resistant (MDR) when they were resistant to at least one agent in three or more antimicrobial categories, extensively resistant (XDR) when they were resistant to at least one agent in all but two or fewer antimicrobial categories, and neither MDR nor XDR when they did not satisfy any of the above criteria [41].

##### Clinical Characteristics

The clinical characteristics were: age (grouped as 18–49 years old, 50–64, and ≥65 years), sex (female/male), comorbidities (obesity, arterial hypertension, heart failure, type 2 diabetes mellitus, asthma, chronic kidney disease (CKD), cancer, and immunosuppression), a variable was generated to group the number of comorbidities (≤2/≥3). We also estimated the total length of stay in the hospital, in mechanic ventilation (MV) and ICU (days).

##### Auxiliary Reference Tests

The following laboratory tests were considered: leukocytes (cells/mm^3^), percentage of lymphocytes (%), platelets (cells/mm^3^), C-reactive protein (CRP; mg/dL), procalcitonin (ng/mL), lactate dehydrogenase (LDH; U/L), total and myocardial creatinine phosphokinase (U/L), aspartate aminotransferase (GOT; U/L), alanine aminotransferase (GPT; U/L), total bilirubin (mg/dL), and creatinine (mg/dL). Oxygen saturation (SatO_2_; ≤80/81–84/85–89/≥90%) and the correlation between oxygen pressure over inspired oxygen fraction (PaO_2_/FiO_2_; <100/100–200/200–300/>300) were evaluated at hospital admission.

##### Complications during ICU Stay

During ICU stay, the complications were: sepsis, septic shock, ARDS, barotrauma, stroke, digestive bleeding, malignant cardiac arrhythmias, and in-hospital cardiac arrest. All were diagnosed according to international criteria [42,43,44,45,46,47,48].

##### Treatment Received

The treatments administered to patients on hospital admission included: antibiotics (ceftriaxone, azithromycin, piperacillin-tazobactam, meropenem, vancomycin, linezolid), corticosteroids (dexamethasone, methylprednisolone), colchicine, tocilizumab, vasopressors, and dialysis.

### 4.4. Statistical Analysis

Categorical variables were described as absolute frequencies and percentages; numerical variables were summarized as mean and standard deviation (SD) or median and interquartile range (IQR) according to whether their distribution was symmetrical or not. The comparison of proportions between categorical variables and the result was performed using the chi2 test or Fisher’s exact test as appropriate. In contrast, for numerical variables, the Student’s T-test was used if there was a standard distribution or, failing that, the Mann–Whitney U test.

To test the hypothesis that healthcare-associated infection was associated with increased hospital mortality in patients with critical COVID-19, Cox proportional hazard models were used to find hazard ratios (HR) and their respective 95% confidence intervals (95% CI). First, clinical variables were tested for associations with outcomes in crude Cox regression models. Then, factors potentially associated with mortality in the crude regression (*p* < 0.2) were included in an adjusted Cox regression model, and the following confounders were added: sex, age, and comorbidities. Compliance with proportionality assumptions was verified using Schoenfeld residuals, and collinearity relationships in the fitted models were assessed. In addition, a secondary analysis was performed to test the hypothesis that healthcare-associated infection was associated with an increased risk of developing acute renal failure, using Poisson regression models with robust variance and finding the relative risks (RR) and their 95% CIs. In the case of AKI, not all medical records recorded the initial date of AKI (less 35%). This is why the Poisson regression with robust variance was used, since it does not use the variable time until the event. The same methodology was used for the crude and adjusted regression as in the Cox model. The linearity of the model was evaluated with each independent variable as well as multicollinearity.

Finally, the survival of patients with bacterial superinfection was described using the Kaplan–Meier method, with death as the event of interest. The log-rank test was used to establish differences in survival. All statistical analyses were performed using Stata^®^ v16 software (StataCorp., College Station, TX, USA).

### 4.5. Ethics

This study was conducted following the Helsinki Declaration of 1975. The institutional research ethics committee approved the Faculty of Health Sciences protocol of the Universidad Privada de Tacna (identification code: 049-FACSA-UI). However, informed consent was not requested due to the retrospective, observational nature of the study.

## 5. Conclusions

There is an overuse of antibiotics from the onset of illness and a high incidence of healthcare-associated infection mainly by XDR pathogens in critically ill patients. Superinfection occurred after one week of ICU stay. It was associated with an increased risk of developing complications such as acute renal failure and significantly raising the risk of death. In this sense, measures should be implemented to promote the rational use of antibiotics in patients with COVID-19, especially those who are critically ill. More studies are needed to evaluate biomarkers that help to guide the indication of antibiotics.

## Figures and Tables

**Table 1:** Clinical and laboratory characteristics of the study population and comparison between survivors and non-survivors.

Variable	All Patients (*n* = 124)	Survivors (*n* = 83)	Non-Survivors (*n* = 41)	*p* Value
**Demographic Characteristics**				
Age, years *	54.47 (±12.03)	54.16 (±11.93)	61 (±10.92)	**0.002** ^a^
Age range, years				
<50 (%)	39 (31.45)	31 (79.49)	8 (20.51)	0.058 ^b^
50–59 (%)	38 (30.65)	26 (68.42)	12 (31.58)	
≥60 (%)	47 (37.90)	26 (55.32)	21 (44.68)	
Sex				0.388 ^b^
Female (%)	22 (17.74)	13 (59.09)	9 (40.91)	
Male (%)	102 (82.26)	70 (68.63)	32 (31.37)	
Number of comorbidities **	1 (1–2)	1 (1–2)	2 (1–3)	**<0.001** ^c^
Comorbidities (%)				
None	18 (14.52)	18 (100)	0 (0)	**0.001** ^b^
1	49 (39.52)	35 (71.43)	14 (28.57)	
2 or more	57 (45.97)	30 (52.63)	27 (47.37)	
Obesity	76 (61.29)	50 (65.79)	26 (34.21)	0.733 ^b^
Diabetes	35 (28.23)	19 (54.29)	16 (45.71)	0.060 ^b^
Hypertension	35 (28.23)	18 (51.43)	17 (48.57)	**0.021** ^b^
Heart failure	8 (6.45)	0 (0)	8 (100)	**<0.001** ^d^
Asthma	24 (19.35)	12 (50)	12 (50)	**0.050** ^b^
Chronic kidney disease	7 (5.65)	0 (0)	7 (100)	**<0.001** ^d^
Cancer	1 (0.81)	1 (100)	0 (0)	0.990 ^d^
Immunosuppression	17 (13.82)	6 (35.29)	11 (64.71)	**0.003** ^b^
Length of hospital stay (days)	19 (14–27.5)	20 (14–26)	18 (14–28)	0.638 ^c^
Time in ICU (days) **	10 (6–16.5)	9 (5–13)	12 (10–24)	**0.001** ^c^
Time in MV (days) **	10 (5–16.5)	7 (4–12)	14 (11–25)	**<0.001** ^c^
Time from ICU admission to superinfection (days) **	8 (6–17)	9 (6–17)	8 (5–15)	0.680 ^c^
**Laboratory Characteristics**				
SatO_2_ (%) **	87 (80–89)	88 (85–90)	85 (77–89)	**0.008** ^c^
Ratio PaO_2_/FiO_2_ **	249.5 (175–294)	264 (215–306)	218 (132–260)	**0.002** ^c^
Leukocytes (cells/mm^3^) **	10,100 (7000–13,000)	10,000 (7000–12,135)	10,700 (7410–16,580)	0.232 ^c^
Percentages of lymphocytes (%) **	7 (5–10)	7 (6–11)	7 (4–9)	0.076 ^c^
Platelets (cells/mm^3^) **	296,000 (234,500–360,000)	318,000 (254,000–369,000)	243,000 (204,000–325,000)	**0.002** ^c^
PCR (mg/dL) **	12.52 (6.62–17.43)	10.56 (4.72–18)	14.23 (10.78–17.25)	**0.032** ^c^
Procalcitonin (ng/mL) **	0.12 (0.03–0.385)	0.07 (0.03–0.24)	0.22 (0.126–0.7)	**<0.001** ^c^
LDH (U/L) **	742.5 (578–984)	700 (565–861)	970 (642–1286)	**0.001** ^c^
CPK-Total (U/L) **	100 (52.5–190.5)	87 (45–161)	161 (79–233)	**0.004** ^c^
CPK-MB (U/L) **	25.25 (20–35)	24.6 (19.6–32)	30 (22–52)	**0.025** ^c^
GOT (U/L) **	50.5 (28–75.5)	50 (28–75)	54 (28–84)	0.614 ^c^
GPT (U/L) **	62.5 (37–116)	66 (44–133)	57 (34–82)	**0.021** ^c^
Total bilirubin (mg/dL) **	0.63 (0.39–0.895)	0.6 (0.37–0.89)	0.63 (0.47–0.96)	0.353 ^c^
Creatinine (mg/dL) **	0.875 (0.73–1.1)	0.84 (0.7–1.05)	0.97 (0.84–1.29)	**0.003** ^c^

* Mean and standard deviation ** Median and interquartile range, ^a^ Student’s t-test for equal variances; ^b^ χ^2^; ^c^ U-Mann–Whitney; ^d^ Fisher’s exact. ICU: intensive care unit, MV: mechanical ventilation, SatO_2_: oxygen saturation, PaO_2_/FiO_2_: ratio of oxygen pressure to inspired oxygen fraction, CRP: C-reactive protein, LDH: lactate dehydrogenase, CPK: creatinine phosphokinase, OGT: aspartate aminotransferase, GPT: alanine aminotransferase. Bold, statistically significant values.

**Table 2:** Complications and treatment received during ICU stay in the study population and comparison between survivors and non-survivors.

Variable	All Patients (*n* = 124)	Survivors (*n* = 83)	Non-Survivors (*n* = 41)	*p* Value
**Complications in ICU**				
Healthcare-associated infection (%)				**<0.001** ^a^
No	74 (59.68)	62 (83.78)	12 (16.22)	
Yes	50 (40.32)	21 (42)	29 (58)	
Type of superinfection				
MV-associated pneumonia (%)				**<0.001** ^a^
No	87 (70.16)	67 (77.01)	20 (22.99)	
Yes	37 (29.84)	16 (43.24)	21 (56.76)	
Catheter-associated bacteremia (%)				0.080 ^b^
No	114 (91.94)	79 (69.30)	35 (30.70)	
Yes	10 (8.06)	4 (40)	6 (60)	
Catheter-associated UTI (%)				0.254 ^b^
No	121 (97.58)	82 (67.77)	39 (32.23)	
Yes	3 (2.42)	1 (33.33)	2 (66.67)	
Bacterial resistance (%)				
No MDR/XDR				0.301 ^b^
No	120 (96.77)	79 (65.83)	41 (34.17)	
Yes	4 (3.23)	4 (100)	0 (0)	
MDR				0.092 ^b^
No	118 (95.16)	81 (68.64)	37 (31.36)	
Yes	6 (4.84)	2 (33.33)	4 (66.67)	
XDR				**<0.001** ^a^
No	84 (67.74)	68 (80.95)	16 (19.05)	
Yes	40 (32.26)	15 (37.50)	25 (62.50)	
Sepsis (%)				**0.002** ^a^
No	41 (33.06)	35 (85.37)	6 (14.63)	
Yes	83 (66.94)	48 (57.83)	35 (42.17)	
Septic shock (%)				**<0.001** ^a^
No	77 (62.10)	64 (83.12)	13 (16.88)	
Yes	47 (37.90)	19 (40.43)	28 (59.57)	
ARDS (%)	124 (100)	83 (66.94)	41 (33.06)	-
Acute kidney injury (%)				**<0.001** ^a^
No	101 (81.45)	79 (78.22)	22 (21.78)	
Yes	23 (18.55)	4 (17.39)	19 (82.61)	
Gastrointestinal bleeding (%)				**0.034** ^b^
No	121 (97.58)	83 (68.60)	38 (31.40)	
Yes	3 (2.42)	0 (0)	3 (100)	
Barotrauma (%)				0.108 ^b^
No	122 (98.39)	83 (68.03)	39 (31.97)	
Yes	2 (1.61)	0 (0)	2 (100)	
Stroke (%)				0.108 ^b^
No	122 (98.39)	83 (68.03)	39 (31.97)	
Yes	2 (1.61)	0 (0)	2 (100)	
Arrhythmia (%)				**<0.001** ^a^
No	117 (94.35)	83 (70.94)	34 (29.06)	
Yes	7 (5.65)	0 (0)	7 (100)	
Cardiac arrest (%)				**<0.001** ^a^
No	94 (75.81)	82 (87.23)	12 (12.77)	
Yes	30 (24.19)	1 (3.33)	29 (96.67)	
Number of complications *	2 (1–3.5)	3 (2–4)	2 (1–3)	**0.001** ^c^
Received antibiotic therapy (%)				0.998 ^b^
No	1 (0.81)	1 (100)	0 (0)	
Yes	123 (99.19)	82 (66.67)	41 (33.33)	
**Antibiotic Therapy Received**				
Ceftriaxone (%)				**0.003** ^a^
No	87 (70.16)	51 (58.62)	36 (41.38)	
Yes	37 (29.84)	32 (86.49)	5 (13.51)	
Azithromycin (%)				**0.029** ^a^
No	115 (92.74)	74 (64.35)	41 (35.65)	
Yes	9 (7.26)	9 (100)	0 (0)	
Piperacillin/Tazobactam (%)				0.280 ^a^
No	69 (55.65)	49 (71.01)	20 (28.99)	
Yes	55 (44.35)	34 (61.82)	21 (38.18)	
Meropenem (%)				**0.036** ^a^
No	93 (75)	67 (72.04)	26 (27.96)	
Yes	31 (25)	16 (51.61)	15 (48.39)	
Vancomycin (%)				0.475 ^a^
No	81 (65.32)	56 (69.14)	25 (30.86)	
Yes	43 (34.68)	27 (62.79)	16 (37.21)	
Linezolid (%)				0.105 ^b^
No	120 (96.77)	82 (68.33)	38 (31.67)	
Yes	4 (3.23)	1 (25)	3 (75)	
Colistin (%)				0.998 ^a^
No	123 (99.19)	82 (66.67)	41 (33.33)	
Yes	1 (0.81)	1 (100)	0 (0)	
Received corticosteroid therapy (%)				0.998 ^b^
No	2 (1.63)	1 (50)	1 (50)	
Yes	121 (98.37)	81 (66.94)	40 (33.06)	
**Corticosteroid Therapy Received**				
Dexamethasone 4 mg (%)				0.598 ^b^
No	120 (96.77)	81 (67.5)	39 (32.5)	
Yes	4 (3.23)	2 (50)	2 (50)	
Dexamethasone 6 mg (%)				**<0.001** ^a^
No	78 (62.9)	42 (53.85)	36 (46.15)	
Yes	46 (37.10)	41 (89.13)	5 (10.87)	
Methylprednisolone 100 mg (%)				**0.001** ^a^
No	57 (45.97)	47 (82.46)	10 (17.54)	
Yes	67 (54.03)	36 (53.73)	31 (46.27)	
Methylprednisolone 500 mg (%)				0.998 ^b^
No	116 (93.55)	78 (67.24)	38 (32.76)	
Yes	8 (6.45)	5 (62.50)	3 (37.50)	
Received colchicine therapy (%)				**0.010** ^a^
No	88 (70.97)	65 (73.86)	23 (23.14)	
Yes	36 (29.03)	18 (50)	18 (50)	
Received tocilizumab therapy (%)				0.111 ^a^
No	98 (79.03)	69 (70.41)	29 (29.59)	
Yes	26 (20.97)	14 (53.85)	12 (46.15)	
Received dialysis therapy (%)				**<0.001** ^a^
No	109 (87.90)	81 (74.31)	28 (25.69)	
Yes	15 (12.10)	2 (13.33)	13 (86.67)	
Received vasopressor therapy (%)				**<0.001** ^a^
No	64 (51.61)	55 (85.94)	9 (14.06)	
Yes	60 (48.39)	28 (46.67)	32 (53.33)	

* Median and interquartile range, ^a^ χ^2^; ^b^ Fisher’s exact; ^c^ U-Mann Whitney. MV: mechanical ventilation, MDR: multidrug-resistant, XDR: extensively resistant, ARDS: acute respiratory distress syndrome; UTI: urinary tract infection. Bold, statistically significant values.

**Table 3:** Clinical and laboratory characteristics of the study population and comparison between those who did and did not develop a healthcare-associated infection.

Variable	No Healthcare-Associated Infection (*n* = 41)	Healthcare-Associated Infection (*n* = 83)	*p* Value
**Demographic Characteristics**			
Age (years) * (%)	53.67 (±12.31)	60.62 (±10.38)	**0.001** ^a^
<50 years (%)	30 (76.92)	9 (23.08)	**0.025** ^b^
50–59 years (%)	21 (55.26)	17 (44.74)	
≥60 years (%)	23 (48.94)	24 (51.06)	
Sex			0.370 ^b^
Female (%)	15 (68.18)	7 (31.82)	
Male (%)	59 (57.84)	43 (42.16)	
Number of comorbidities **	1 (1–2)	1 (1–3)	0.422 ^c^
No comorbidity (%)	13 (72.22)	5 (27.78)	0.449 ^b^
Only 1 (%)	27 (55.10)	22 (44.90)	
More than 2 (%)	34 (59.65)	23 (40.35)	
Obesity (%)	46 (60.53)	30 (39.47)	0.808 ^b^
Diabetes (%)	20 (57.14)	15 (42.86)	0.718 ^b^
Hypertension (%)	19 (54.29)	16 (45.71)	0.443 ^b^
Heart failure (%)	5 (62.50)	3 (37.50)	0.998 ^d^
Asthma (%)	15 (62.50)	9 (37.50)	0.754 ^b^
Chronic kidney disease (%)	1 (14.29)	6 (85.71)	**0.017** ^d^
Cancer (%)	1 (100)	0 (0)	0.998 ^d^
Immunosuppression (%)	9 (52.94)	8 (47.06)	0.512 ^b^
Length of hospital stay (days) **	17.5 (13–23)	21.5 (16–32)	**0.003** ^c^
Time in ICU (days) **	8 (4–12)	13 (10–23)	**0.001** ^c^
Time in MV (days) **	7 (4–11)	14.5 (10–27)	**<0.001** ^c^
**Laboratory Characteristics**			
SatO_2_ (%) **	87.5 (84–90)	85 (78–89)	0.055 ^c^
PaO_2_/FiO_2_ ratio **	261 (220–307)	224.5 (136–276)	**0.002** ^c^
Leukocytes (cells/mm^3^) **	9990 (7000–11,000)	11,865 (8590–16,810)	**0.011** ^c^
Percentage of lymphocytes (%) **	8 (6–11)	6.2 (3.7–10)	**0.042** ^c^
Platelets (cells/mm^3^) **	308,500 (245,000–362,000)	276,000 (210,000–356,000)	0.134 ^c^
CRP (mg/dL) **	11.5 (5.22–17.35)	13.59 (9.92–17.95)	0.189 ^c^
Procalcitonin (ng/mL) **	0.06 (0.03–0.17)	0.3 (0.126–0.69)	**<0.001** ^c^
LDH (U/L) **	691.5 (552–854)	853 (639–1160)	**0.001** ^c^
CPK-Total (U/L) **	87 (47–161)	132.5 (66–225)	**0.028** ^c^
CPK-MB (U/L) **	25 (19.9–35)	27.75 (20–40)	0.469 ^c^
Total bilirubin (mg/dL) **	0.68 (0.39–0.90)	0.615 (0.39–0.83)	0.624 ^c^
Creatinine (mg/dL) **	0.84 (0.7–0.98)	0.96 (0.79–1.25)	**0.002** ^c^
**Treatment Administered**			
Received antibiotic therapy (%)			0.998 ^d^
No	1 (100)	0 (0)	
Yes	73 (59.35)	50 (40.65)	
Antibiotic therapy received			
Ceftriaxone (%)			**0.006** ^b^
No	45 (51.72)	42 (48.28)	
Yes	29 (78.38)	8 (21.62)	
Azithromycin (%)			0.083 ^d^
No	66 (57.39)	49 (42.61)	
Yes	8 (88.89)	1 (11.11)	
Piperacillin/Tazobactam (%)			0.076 ^b^
No	46 (66.67)	23 (33.33)	
Yes	28 (50.91)	27 (49.09)	
Meropenem (%)			0.291 ^b^
No	58 (62.37)	35 (37.63)	
Yes	16 (51.61)	15 (48.39)	
Vancomycin (%)			0.799 ^b^
No	49 (60.49)	32 (39.51)	
Yes	25 (58.14)	18 (41.86)	
Linezolid (%)			0.998 ^d^
No	72 (60)	48 (40)	
Yes	2 (50)	2 (50)	
Colistin (%)			0.998 ^d^
No	73 (59.35)	50 (40.65)	
Yes	1 (100)	0 (0)	
Received corticosteroid therapy (%)			0.085 ^d^
No	0 (0)	2 (100)	
Yes	73 (60.33)	48 (39.67)	
**Corticotherapy Received**			
Dexamethasone 4 mg (%)			0.647 ^d^
No	71 (59.17)	49 (40.83)	
Yes	3 (75)	1 (25)	
Dexamethasone 6 mg (%)			**0.013** ^b^
No	40 (51.28)	38 (48.72)	
Yes	34 (73.91)	12 (26.09)	
Methylprednisolone 100 mg (%)			0.067 ^b^
No	39 (68.42)	18 (31.58)	
Yes	35 (52.24)	32 (47.76)	
Methylprednisolone 500 mg (%)			0.266 ^d^
No	71 (61.21)	45 (38.79)	
Yes	3 (37.50)	5 (62.50)	
Received colchicine therapy (%)			0.071 ^b^
No	57 (64.77)	31 (35.23)	
Yes	17 (47.22)	19 (52.78)	
Received tocilizumab therapy (%)			0.258 ^b^
No	61 (62.24)	37 (37.76)	
Yes	13 (50)	13 (50)	
Received dialysis therapy (%)			**0.001** ^b^
No	71 (65.14)	38 (34.86)	
Yes	3 (20)	12 (80)	
Received vasopressor therapy (%)			**0.004** ^b^
No	46 (71.88)	18 (28.13)	
Yes	28 (46.67)	32 (53.33)	

* Mean and standard deviation ** Median and interquartile range; ^a^ Student’s t-test for equal variances; ^b^ χ^2^; ^c^ U-Mann–Whitney; ^d^ Fisher’s exact. ICU: intensive care unit, MV: mechanical ventilation, SatO_2_: oxygen saturation, PaO_2_/FiO_2_: ratio of oxygen pressure to inspired oxygen fraction, CRP: C-reactive protein, LDH: lactate dehydrogenase, CPK: creatinine phosphokinase. Bold, statistically significant values.

**Table 4:** Cox regression analysis to evaluate predictors of mortality in patients hospitalized for critical COVID-19.

	Crude Model		Adjusted Model	
**Variable**	**cHR (95% CI)**	***p*** **Value**	**aHR (95% CI)**	***p*** **Value**
**Healthcare-associated infection**	2.216 (1.12–4.39)	0.023	**2.733 (1.33–5.60)**	**0.006**
Age ≥ 60 years	1.045 (0.45–2.40)	0.917	0.937 (0.32–2.72)	0.905
Male	0.685 (0.33–1.44)	0.320	0.582 (0.35–1.80)	0.582
**Obesity**	1.581 (0.82–3.02)	0.166	**2.605 (1.11–6.07)**	**0.027**
Diabetes	1.804 (0.95–3.40)	0.068	1.903 (0.90–4.00)	0.090
Hypertension	1.375 (0.73–2.57)	0.319	1.172 (0.51–2.65)	0.703
Asthma	1.461 (0.74–2.88)	0.273	1.463 (0.66–3.23)	0.346
Heart failure	2.670 (1.22–5.83)	0.014	**4.701 (1.58–13.97)**	**0.005**
PaO_2_/FiO_2_				
**+300**	Ref		Ref	
**200–300**	2.559 (0.75–8.66)	0.131	**4.256 (1.14–15.80)**	**0.044**
**100–200**	3.917 (1.05–14.51)	0.041	**3.796 (1.01–14.47)**	**0.050**
**−100**	5.011 (1.37–18.25)	0.015	**5.503 (1.34–22.52)**	**0.018**

cHR: crude hazard ratio, aHR: adjusted hazard ratio, PaO_2_/FiO_2_: ratio of oxygen pressure to inspired oxygen fraction. In-hospital mortality was adjusted for sex, age, obesity, diabetes, hypertension, asthma, heart failure, and PaO_2_/FiO_2_ less than 300. The proportionality of the multivariate model had a *p* value of *p* = 0.8452. Ref, reference group. Bold, significant values.

**Table 5:** Poisson regression analysis to evaluate risk factors for acute kidney injury in patients hospitalized for critical COVID-19.

	**Crude Model**		**Adjusted Mode**	
**Variable**	**cRR (95% CI)**	***p*** **Value**	**aRR (95% CI)**	***p*** **Value**
**Healthcare-associated infection**	**3.594 (1.60–8.05)**	**0.002**	**3.093 (1.42–6.72)**	**0.004**
Age ≥ 60 years	1.797 (0.75–4.30)	0.188	1.370 (0.50–3.69)	0.533
Male	1.509 (0.49–4.64)	0.472	1.935 (0.56–6.59)	0.291
Obesity	1.533 (0.68–3.43)	0.298	2.537 (1.16–5.54)	0.019
Diabetes	1.525 (0.73–3.16)	0.257	1.091 (0.49–2.40)	0.828
Hypertension	2.151 (1.06–4.35)	0.033	1.504 (0.66–3.40)	0.327
Asthma	1.715 (0.80–3.67)	0.165	2.358 (1.19–4.96)	0.024
Heart failure	2.9 (1.29–6.47)	0.009	2.045 (0.67–6.21)	0.207
PaO_2_/FiO_2_				
+300	Ref		Ref	
200–300	2.306 (0.54–9.74)	0.256	1.946 (0.51–7.35)	0.326
100–200	2.052 (0.37–11.18)	0.406	1.169 (0.23–5.92)	0.850
−100	6.117 (1.46–25.55)	0.013	3.972 (0.97–16.22)	0.055

cRR: crude relative risk; aRR: adjusted relative risk; PaO_2_/FiO_2_: ratio of oxygen pressure to inspired oxygen fraction. Acute renal failure was adjusted for sex, age, obesity, diabetes, hypertension, asthma, heart failure, and PaO_2_/FiO_2_ less than 300. The model assumptions had a VIF of 2.08. Ref, reference group. Bold, significant values.

## Data Availability

Available upon reasonable request.

## Supplementary Materials

The following are available online at https://www.mdpi.com/article/10.3390/antibiotics10080959/s1, Tables S1: Pathogens found in the cultures of the study population.; Table S2: Description of the antibiotic resistance profile of Acinetobacter Baumannii present in the study cultures (n = 27).; Table S3: Description of the antibiotic resistance profile of Pseudomonas aeruginosa present in the study cultures (n = 11).
