# Supplementary material for: Antibiotic Use and Fatal Outcomes among Critically Ill Patients with COVID-19 in Tacna, Peru
Source: Antibiotics (Basel). 2021 Aug 9;10(8):959. doi: 10.3390/antibiotics10080959 (PMC8388967; doi:10.3390/antibiotics10080959)
Supplement: Supplementary file 1 [file antibiotics-10-00959-s001.zip › antibiotics-1311655-supplementary.pdf]

antibiotics

Supplementary Materials:

Article

Antibiotic Use and Fatal Outcomes among Critically Ill Patients with COVID-19 in Tacna, Peru

Cesar Copaja-Corzo [1], Miguel Hueda-Zavaleta [1,2,*], Vicente A. Benites-Zapata [3] and Alfonso J. Rodriguez-Morales [4,5,*]

[1] Faculty of Health Sciences, Universidad Privada de Tacna, Tacna 23003, Peru; cescopajac@upt.pe
[2] Hospital III Daniel Alcides Carrión EsSalud, Tacna 23000, Peru
[3] Universidad San Ignacio de Loyola, Unidad de Investigación para la Generación y Síntesis de Evidencias en Salud, Lima 15024, Peru; vbenites@usil.edu.pe
[4] Masters of Clinical Epidemiology and Biostatistics, Universidad Cientifica del Sur, Lima 15046, Peru
[5] Grupo de Investigación Biomedicina, Faculty of Medicine, Fundación Universitaria Autónoma de las Américas, Belmonte, Pereira, Risaralda 660003, Colombia
* Correspondence: miguel.hueda@essalud.gob.pe (M.H.-Z.); arodriguezmo@cientifica.edu.pe (A.J.R.-M.)

Table S1. Pathogens found in the cultures of the study population.

Antibiotic	Total (*n* = 50)	VAP (*n* = 37)	CAB (*n* = 10)	CAUTI (*n* = 3)
Acinetobacter baumannii	27 (54)	25 (67.57)	2 (20)	0 (0)
Pseudomonas aeuriginosa	11 (22)	7 (18.92)	1 (10)	3 (100)
Staphylococcus epidermidis	3 (6)	0 (0)	3 (30)	0 (0)
Klebsiella pneumoniae	4 (8)	3 (8.11)	1 (10)	0 (0)
Stenotrophomona maltophilia	2 (4)	1 (2.7)	1 (10)	0 (0)
Klebsiella oxytoca	1 (2)	1 (2.7)	0	0 (0)
Enterococcus faecalis	1 (2)	0 (0)	1 (10)	0 (0)
Staphylococcus haemolyticus	1 (2)	0(0)	1 (10)	0 (0)

VAP: Ventilator-associated pneumonia, CAB: Catheter-associated bacteremia, CAUTI: Catheter-associated urinary tract infection.

Table S2. Description of the antibiotic resistance profile of *Acinetobacter Baumannii* present in the study cultures (*n* = 27).

Antibiotic	Susceptible, *n* (%)	Resistant, *n* (%)	Intermediate. *n* (%)
Amikacin	2 (7.41)	25 (92.59)	0 (0)
Ampicillin/Sulbactam	9 (33.33)	7 (25.93)	11 (40.74)
Cefepime	3 (11.11)	24 (88.89)	0 (0)
Ceftazidime	1 (3.70)	22 (81.48)	4 (14.81)
Ciprofloxacin	1 (3.70)	26 (96.30)	0 (0)
Gentamicin	1 (3.70)	26 (96.30)	0 (0)
Imipenem	1 (3.70)	26 (96.30)	0 (0)
Levofloxacin	1 (3.70)	26 (96.30)	0 (0)
Meropenem	1 (3.70)	26 (96.30)	0 (0)
Tobramycin	2 (7.41)	25 (92.59)	0 (0)

Table S3. Description of the antibiotic resistance profile of *Pseudomonas aeruginosa* present in the study cultures (n = 11).

**Antibiotic**	**Susceptible, *n* (%)**	**Resistant, *n* (%)**	**Intermediate, *n* (%)**
Amikacin	0 (0)	11 (100)	0 (0)
Aztreonam	1 (9.09)	10 (90.91)	0 (0)
Cefepime	0 (0)	11 (100)	0 (0)
Ceftazidime	0 (0)	11 (100)	0 (0)
Ciprofloxacin	0 (0)	11 (100)	0 (0)
Colistin	11 (100)	0 (0)	0 (0)
Gentamicin	0 (0)	10 (90.91)	1 (9.09)
Imipenem	0 (0)	11 (100)	0 (0)
Levofloxacin	0 (0)	11 (100)	0 (0)
Meropenem	0 (0)	11 (100)	0 (0)
Piperacillin/Tazobactam	7 (63.64)	1 (9.09)	3 (27.27)
Tobramycin	0 (0)	7 (100)	0 (0)
